# Progression of Dupuytren Contracture: A Randomized Controlled Trial Comparing Surgery, Needle Fasciotomy, and Collagenase Injection

**DOI:** 10.1097/PRS.0000000000012666

**Published:** 2025-12-04

**Authors:** Venla-Linnea Karjalainen, Janne Soikkeli, Mikko P. Räisänen, Olli V. Leppänen, Aleksi Reito, Susanna Stjernberg-Salmela, Rachelle Buchbinder, Teemu Karjalainen

**Affiliations:** Tampere, Oulu, Kuopio, and Helsinki, Finland; and Melbourne, Australia; From the 1 Faculty of Medicine and Health Technology, Tampere University; 2 Department of Hand Surgery and Orthopedics, Oulu University Hospital; 3 Department of Orthopedics, Traumatology and Hand Surgery, Kuopio University Hospital; Departments of 4 Hand Surgery and Microsurgery; 5 Orthopedics, Tampere University Hospital; 6 Department of Musculoskeletal and Plastic Surgery, Helsinki University Hospital; 7 Musculoskeletal Health and Wiser Health Care Units, School of Public Health and Preventive Medicine, Monash University.

## Abstract

**Background:**

Dupuytren contracture can be treated by surgery, needle fasciotomy, or collagenase injection, but how these treatments affect disease progression in both treated and untreated fingers is poorly understood. This study aimed to compare the progression of Dupuytren contracture across these 3 treatments over a 2-year follow-up and identify patients at highest risk for progression and retreatment.

**Methods:**

This finger-level analysis used data from DETECT (Dupuytren Treatment Effectiveness Trial) and included 302 participants with 423 initially treated fingers. Progression was defined as an increase in contracture angle between 3 months and 2 years after treatment. Analyses included treated, adjacent untreated, and all untreated fingers. The authors used linear regression models to identify risk factors for progression and retreatment.

**Results:**

Of the 302 participants, 279 (92%) completed 3-month contracture measurements and 274 (91%) completed 2-year contracture measurements. In treated fingers, surgery resulted in less progression than needle fasciotomy (mean difference, 9.7 degrees [95% CI, 3.7 to 16]) and collagenase injection (mean difference, 6.0 degrees [95% CI, 0.1 to 12]), and fewer retreatments. No between-group differences were observed in adjacent untreated or all untreated fingers. Younger age at treatment, smoking, dominantly affected proximal interphalangeal joint, and involvement of the little finger were associated with increased risk of progression or retreatment.

**Conclusions:**

Surgery offers more durable results in treated fingers compared with percutaneous methods, although progression and retreatment rates in untreated fingers are similar across all treatment groups. Patients at higher risk for progression (ie, young smokers with affected little fingers) may benefit most from surgery.

Dupuytren contracture is a hereditary condition characterized by the gradual tightening of cords within the palmar fascia, resulting in the inability to extend one or more fingers fully,^1^ impairing hand function and limiting daily activities, such as picking up objects.^2^ Predominantly affecting individuals of middle to advanced age, its prevalence is estimated to lie between 1% and 30%, with the highest prevalence rates among people of northern European descent.^3^ Dupuytren contracture lacks a definitive cure^4^; treatment aims to alleviate symptoms by addressing the contracture through surgery, needle fasciotomy, or collagenase injection, but the contracture frequently recurs and often requires retreatment.

Surgery carries a lower recurrence risk in treated fingers compared with needle fasciotomy and collagenase injection.^5^ However, most studies focus solely on recurrence as a binary outcome in the treated finger, leaving a significant gap in understanding the broader impact of treatment on disease progression. Specifically, the effect of the treatment options on the gradual progression of the disease in both treated and adjacent untreated fingers remains unexplored. Wider excision of pathological tissue with surgery, as opposed to division of the contracted fascial cord with percutaneous treatments, could theoretically protect against the progression of the disease in other fingers. Conversely, the greater tissue trauma caused by surgery could in theory trigger disease activation in adjacent fingers, leading to faster progression in previously unaffected fingers.^4,6–10^

This study presents a secondary, finger-level analysis of data from DETECT (Dupuytren Treatment Effectiveness Trial), a randomized controlled trial primarily aimed at comparing the effectiveness of surgery, needle fasciotomy, and collagenase. A primary 2-year analysis of DETECT, which focused exclusively on participant-level outcomes and included only treated fingers, has been published.^11^ Initial outcomes were comparable across all treatment groups; however, by the 2-year follow-up, success rates were sustained in the surgery group but had declined in the needle fasciotomy and collagenase groups, even after accounting for retreatments.^11^

This secondary analysis aimed to compare the progression of Dupuytren contracture, defined as an increase in contracture angle after primary treatment, among 3 treatment groups (surgery, needle fasciotomy, and collagenase injection) in both treated and untreated fingers. We also explored factors associated with progression and the risk of retreatment in treated fingers to identify factors that could potentially affect treatment choice.

## PATIENTS AND METHODS

### Design and Setting

DETECT is a multicenter, randomized, outcome assessor–blinded, 3-armed (1:1:1) parallel superiority trial comparing surgery, needle fasciotomy, and collagenase injection in participants with treatment-naive Dupuytren contracture. The trial includes a 10-year follow-up, with the primary time point set at 5 years. This article uses finger-level data from prespecified early secondary time points (3 months and 2 years).

In the surgery group, participants receive surgical interventions for all recurrences; in the percutaneous groups, participants may opt for another percutaneous treatment or surgery for retreatments. The trial was registered on clinicaltrials.gov (NCT03192020) before recruitment commenced, and the complete trial protocol has been published.^12^

The study centers comprised 6 national secondary and tertiary hospitals in Finland operating within a universal health care system. The study protocol gained approval from the institutional review board of Tampere University Hospital and the Finnish Medicines Agency. An independent party monitored the study sites both before and during the trial.

### Participants

A hand surgeon investigator screened individuals referred for Dupuytren contracture treatment at each study center. Upon confirming eligibility, the investigator obtained written informed consent, and the study site research nurse collected baseline data during the initial visit.

The inclusion and exclusion criteria are detailed in Table 1. Exclusions encompassed any other condition affecting the function of the affected fingers as well as a severe total contracture (>135 degrees).

**Table 1.** Inclusion and Exclusion Criteria

Inclusion Criteria	Exclusion Criteria
Age 18 years or older	Age 80 years or older
At least 20 degrees combined extension deficit in the metacarpophalangeal or proximal interphalangeal joint, or both, in fingers 2 through 5	Total passive extension deficit greater than 135 degrees
A palpable cord	Previous fracture in the finger affecting range of motion of the metacarpophalangeal or proximal interphalangeal joint
Provision of informed consent	Recurrent contracture, rheumatoid arthritis, neurologic condition affecting finger function, pregnancy, or breastfeeding
Ability to fill out the Finnish versions of the questionnaires	Contraindication for collagenase

### Randomization

We used a centralized allocation system to ensure concealment of treatment allocation from the investigators. The computer-generated random sequence was created by an investigator who did not participate in the recruitment or treatment of the participants throughout the study (A.R.). Following consent and recording of baseline data in the database, the investigator contacted the coordinating research nurse by telephone for the allocation code. None of the investigators involved in recruitment or follow-up visits had access to the randomization list. The participants were allocated in a 1:1:1 ratio using a random block size, stratified by the joint with the greatest contracture angle: the metacarpophalangeal joint (MPJ) or proximal interphalangeal joint (PIPJ).

### Blinding

Participants were not blinded to their treatment allocation. Blinded outcome assessors, who had no other role in the study, measured contracture angles with the participant’s hand covered by an opaque rubber glove during measurements. Participants were specifically instructed not to disclose their treatment allocation to the assessor.

### Interventions

Board-certified specialists in hand surgery with 2 to 17 years of experience (mean 8.2 years) performed all procedures, including percutaneous treatments. Surgery took place in the operating theater. Percutaneous treatments were done in the outpatient clinic. Participants allocated to a percutaneous treatment typically received it on the same day as recruitment; those assigned to surgery were scheduled for elective surgery.

The surgery group received limited fasciectomy, where cords limiting the extension were excised, safeguarding digital nerves and arteries.^12^ For the needle fasciotomy group, the surgeon injected local anesthesia in the skin overlying the cord and then divided it in 1 to 3 levels using the tip of an 18-gauge needle, leaving only puncture holes in the skin. For the collagenase group, injection without local anesthesia was followed by finger extension manipulation under local anesthesia 1 to 3 days later in the clinic. Collagenase injection could be repeated up to 3 times if needed or if multiple fingers required treatment.

All participants received standardized instructions for postoperative self-administered rehabilitation exercises. Night splints were used at the surgeon’s discretion. Surgery participants had a bulky dressing for 2 to 3 days and resumed free use after the wound healed, typically within 2 to 3 weeks. Participants in the surgery group were offered only surgery for reinterventions. Percutaneous treatment recipients were advised to use the hand as tolerated after the procedure. They could opt for another percutaneous treatment or surgery if unsuccessful at the 3-month assessment. All participants could contact their study center for further treatment needs of recurrence or progression of contracture in nontreated fingers.

### Outcomes

The primary outcome of the analysis was the progression of Dupuytren contracture, defined as an increase in the contracture angle between the 3-month and 2-year follow-up points (with a higher angle indicating faster progression). The 3-month time point is used as the starting point for progression because it represents the earliest measured time point when the initial treatment effects have stabilized. Assessors who were blinded to treatment allocation measured the contracture angles using a handheld goniometer. The angles were recorded as the sum of the MPJ, PIPJ, and distal interphalangeal joint contractures when the finger was fully passively extended by the assessor. Hyperextension was recorded as 0 degrees.

Retreatment was defined as a treatment to any finger after the initial intervention regardless of whether the finger was initially treated. The study center research nurse checked patient case notes for retreatments for all participants, including those who did not participate in the 2-year follow-up. All randomized fingers were thus included in the analyses regarding retreatments.

The study site research nurse also asked whether any serious adverse events had occurred. Minor transient adverse events, which typically do not require medical attention and are expected in all individuals undergoing surgery, were not recorded.

### Statistical Analysis

The unit of analysis was the finger. Progression was compared separately for initially treated fingers, adjacent untreated fingers (fingers that were initially untreated but directly adjacent to treated ones), and all initially untreated fingers. Contractures of the thumb were not included in this study.

We present the descriptive statistics as mean ± SD, median with interquartile range, or count with percentage. Fingers that underwent retreatment before the 2-year follow-up were excluded from the contracture progression analysis. However, as part of a sensitivity analysis, we also conducted the progression analysis including the fingers that underwent retreatment.

For the analysis of progression, we used a linear mixed-effects model. The main exposure was group allocation and fixed effects included baseline contracture, study center, time × group interaction, and dominantly affected joint (MPJ or PIPJ). Fingers were nested within participants as a random effect. The treatment effects were expressed as adjusted mean differences with 95% CIs. Model fit was assessed using marginal and conditional R^2^, representing the variance explained by the fixed effects and by the full model including random effects, respectively.^13,14^

To compare retreatments between groups, we created Kaplan-Meier survival curves, using the recruitment date as the start point and the retreatment date as the end point. Censored observations were incorporated to account for both dropouts (including deaths) and fingers that did not require retreatment within 2 years after trial recruitment. Differences between treatment groups were assessed using the log-rank test with Bonferroni adjustment applied for multiple pairwise comparisons.

For the analysis of prognostic factors, we included only initially treated fingers, using a linear model and interpreting the untransformed regression coefficients as the effects. Treatment group was included as a fixed factor in each model. The potential prognostic factors included age, sex, duration of contracture, self-reported family history of Dupuytren contracture, smoking status, baseline contracture, little finger being affected, dominantly affected joint (MPJ or PIPJ), number of treated fingers, diabetes, and alcohol consumption. After univariable analysis, we entered variables that were associated with progression in a linear multivariable model to identify variables predicting progression. To analyze prognostic factors for retreatment, we used a similar approach using a logistic regression model, and report odds ratios with 95% CIs. Model fit for the multivariable linear model was assessed using adjusted R^2^, and model performance for the multivariable logistic regression model was evaluated using the area under the receiver operating characteristic curve.

We assessed statistical significance using 95% CIs and considered *P* values less than 0.05 statistically significant.

All analyses were done using the lme4, stats, emmeans, survival, MuMIn, and pROC packages in R (version 4.4.1) (R Foundation for Statistical Computing).^15–20^

## RESULTS

**Fig. 1.**
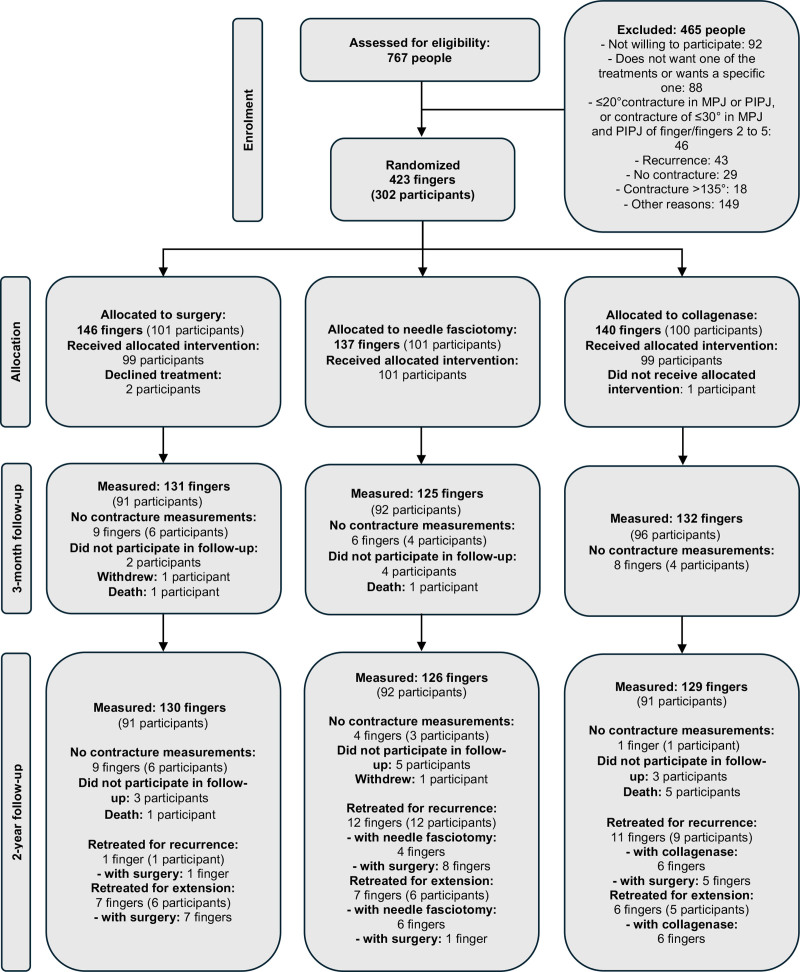
Consolidated Standards of Reporting Trials flow diagram.

We recruited 302 participants with (423 fingers) initially treated between September 15, 2017, and February 2, 2021 (Fig. 1).

Some participants declined on-site assessments, primarily due to the COVID-19 pandemic, but agreed to provide self-reported outcomes through email. Complete data were available for 279 participants (92%) at 3 months, with an additional 14 participants (4.6%) providing self-reported outcomes. At the 2-year mark, complete data were available for 274 participants (91%), with 10 additional participants (3.3%) providing self-reported outcomes. There were no significant differences in completion rates between the groups. The median waiting time for surgery was 81 days (range, 2 to 484 days).

Table 2 displays the baseline characteristics across groups.

**Table 2.** Participant Characteristics^a^

Characteristics	Surgery (*n* = 101)	Needle Fasciotomy (*n* = 101)	Collagenase (*n* = 100)
Age, yrs	65 ± 7.6	66 ± 7.4	65 ± 8.2
Female	16 (16)	23 (23)	19 (19)
Duration of symptoms, yrs	7.6 ± 6.1	8.4 ± 6.5	8.1 ± 6.8
Family history of Dupuytren contracture	40 (40)	41 (41)	34 (34)
Smoking	23 (23)	16 (16)	24 (24)
Dominant hand affected	83 (82)	75 (74)	78 (78)
Diabetes	23 (23)	20 (20)	22 (22)
Alcohol consumption^b^	4 (1–10)	3 (0–7)	4 (1–10)
Hypertension	40 (40)	40 (40)	44 (44)
Hypercholesterolemia	31 (31)	29 (29)	27 (27)
Coronary artery disease	9 (9)	7 (7)	5 (5)
Trust in treatment efficacy^c^	95 (94)	86 (85)	84 (84)
Ledderhose disease	12 (12)	13 (13)	11 (11)
Peyronie disease	1 (1)	4 (5)	1 (1)
Garrod pads	4 (4)	2 (2)	4 (4)
PIPJ contracture type^d^			
Right hand			
No contracture	63 (62)	61 (60)	55 (55)
Static	28 (28)	34 (34)	33 (33)
Dynamic	10 (10)	6 (6)	12 (12)
Left hand			
No contracture	63 (62)	67 (66)	60 (60)
Static	32 (32)	31 (31)	29 (29)
Dynamic	6 (6)	3 (3)	11 (11)
Employment			
Unemployed or retired^e^	65 (64)	75 (74)	66 (66)
Blue-collar	20 (20)	16 (16)	19 (19)
White-collar	16 (16)	10 (10)	15 (15)
QuickDASH score (0 to 100; lower is better)	17.9 ± 16	14.1 ± 12	15.6 ± 13
VAS function score (0 to 100 mm; higher is better)	67.0 ± 21	71.7 ± 18	70.9 ± 19
EQ-5D-EUR index score^f^ (0 to 1; higher is better)	0.82 ± 0.17	0.86 ± 0.16	0.85 ± 0.15
EuroQol VAS (0 to 100; higher is worse)	80 ± 17	81 ± 17	81 ± 13
Total flexion, degrees	242 ± 39	237 ± 30	244 ± 30
Contracture, degrees			
MPJ	37 ± 24	40 ± 23	36 ± 23
PIPJ	27 ± 25	27 ± 26	27 ± 24
Total contracture,^g^ degrees	63 ± 29	65 ± 26	61 ± 25
MPJ dominantly affected	62 ± 30	61 ± 24	58 ± 26
PIPJ dominantly affected	65 ± 26	73 ± 28	66 ± 23

VAS, visual analog scale.

a Data are presented as mean ± SD, *n* (%), or median (interquartile range).

b Standardized drinks per week.

c Percentage of participants who answered “totally agree” or “partially agree” to “the treatment you are about to receive is effective for your condition.”

d Definition for dynamic PIPJ contracture: the PIPJ had 20 or greater degrees of contracture but could be fully extended when MPJ was held in 90 degrees of flexion. Static contracture could not be fully extended.

e Retired at the 3-month follow-up.

f European reference values for the European Quality of Life–5 Dimensions–3 Level index score.

g Sum of contracture for MPJ, PIPJ, and distal interphalangeal joint.

### Comparison of Treatments

**Fig. 2.**
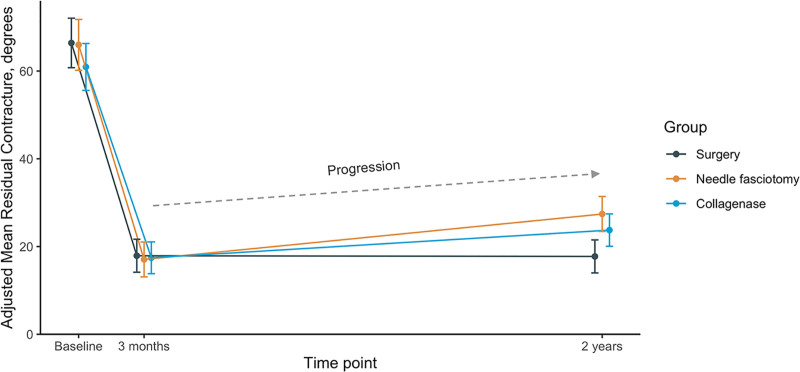
Line graphs of adjusted mean residual contractures showing contracture progression in treated groups. Vertical lines represent 95% CIs.

In the initially treated fingers, between 3 months and 2 years after surgery, surgery resulted in 9.7 degrees slower progression (95% CI, 3.7 to 16) compared with fingers treated with needle fasciotomy and 6.0 degrees slower progression (95% CI, 0.1 to 12) compared with collagenase (Fig. 2). For the linear mixed-effects model, the marginal R^2^ was 0.27 and the conditional R^2^ was 0.66.

**Fig. 3.**
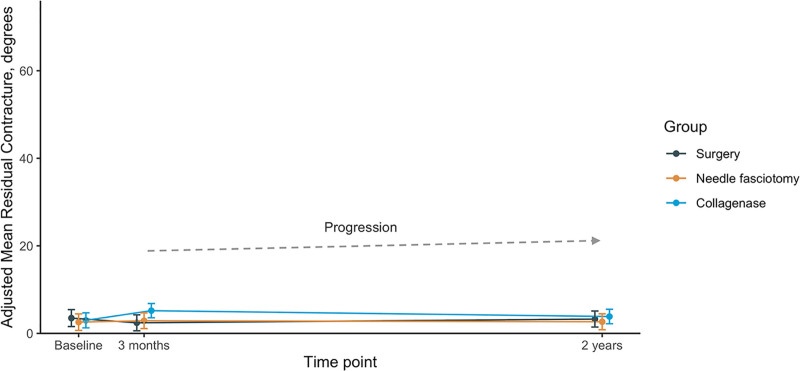
Line graphs of adjusted mean residual contractures showing contracture progression in adjacent untreated fingers. Vertical lines represent 95% CIs.

**Fig. 4.**
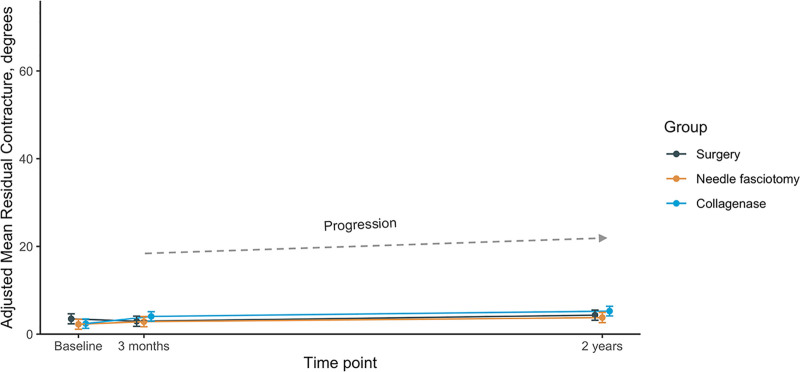
Line graphs of adjusted mean residual contractures showing contracture progression in all untreated fingers. Vertical lines represent 95% CIs.

In adjacent untreated and all initially untreated fingers, progression was comparable between treatment groups (Figs. 3 and 4). (**See Table, Supplemental Digital Content 1**, which details raw means and adjusted pairwise treatment effects for residual contractures at the 3-month and 2-year follow-up points, https://links.lww.com/PRS/I559.) Including fingers that underwent retreatment in these analyses did not influence the results considerably.

**Fig. 5.**
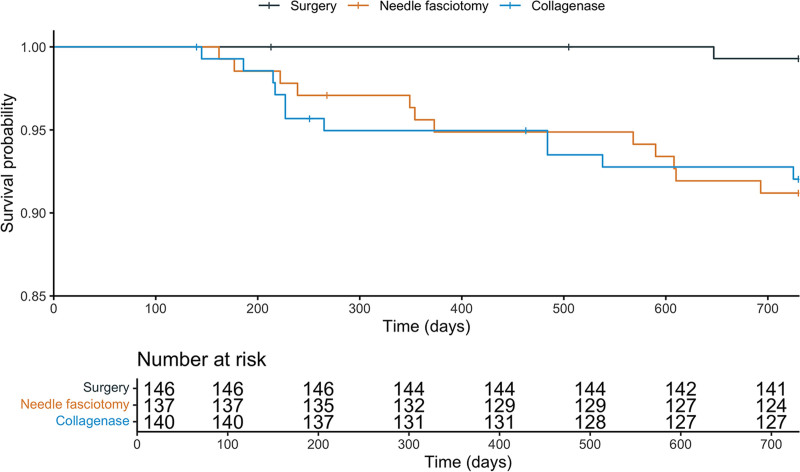
Kaplan-Meier curves for retreatments in initially treated fingers.

**Fig. 6.**
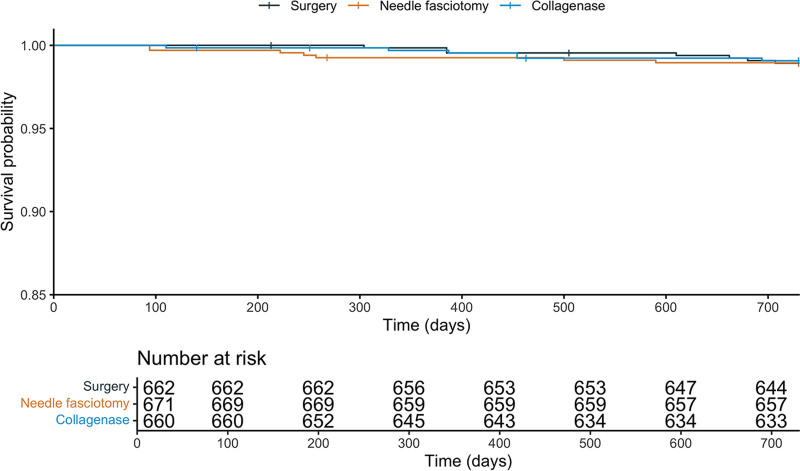
Kaplan-Meier curves for retreatments in initially untreated fingers.

The risk of retreatment was lowest in the surgery group. Less than 1% (1 of 146) of initially treated fingers in the surgery group required retreatment, compared with 9% (12 of 137) in the needle fasciotomy group and 8% (11 of 140) in the collagenase group (Fig. 5). Pairwise log-rank tests indicated a lower retreatment risk for surgery versus needle fasciotomy (*P* = 0.004) and surgery versus collagenase (*P* = 0.007), whereas the needle fasciotomy and collagenase groups did not differ (*P* = 1.00). For initially untreated fingers, the risk for retreatment was comparable between the groups (Fig. 6).

Three serious adverse events were observed: 1 arterial injury and 1 digital nerve injury in the surgery group, and 1 flexor tendon rupture in the needle fasciotomy group.

### Prognostic Factors

In univariable models, age, smoking, baseline contracture, little finger being affected, and the number of fingers affected were associated with progression (measured in degrees) in treated fingers. Having the little finger affected and the PIPJ being the dominantly affected joint were associated with retreatment in initially treated fingers (Table 3). The area under the receiver operating characteristic curve for the multivariable logistic regression model was 0.798.

**Table 3.** Prognostic Factors for Progression and Retreatment in Initially Treated Fingers

Progression	Intercept	β Coefficient	95% CI	*P*
Age^a^	43.1	−0.49	−0.76 to −0.22	0.00
Sex	10.1	1.43	−3.74 to 6.60	0.59
Duration of contracture	1.35	−0.17	−0.46 to 0.12	0.25
Family history of Dupuytren contracture	9.38	2.31	−1.57 to 6.19	0.24
Smoking^a^	9.69	6.83	1.77 to 11.88	0.01
Baseline contracture^a^	5.02	0.09	0.01 to 0.16	0.02
Little finger affected^a^	7.80	5.00	1.22 to 8.77	0.01
PIPJ dominantly affected	−1.02	2.62	−1.48 to 6.72	0.21
Number of treated fingers^a^	5.08	−2.84	−4.80 to −0.89	0.00
Diabetes	0.38	−2.01	−6.90 to 2.89	0.42
Alcohol consumption	−0.10	0.01	−0.21 to 0.23	0.90
Multivariable model	24.3			
Age^a^		−0.42	−0.69 to −0.15	0.00
Smoking^a^		5.34	0.36 to 10.3	0.03
Baseline contracture		0.06	−0.02 to 0.13	0.13
Little finger affected		3.27	−0.60 to 7.14	0.10
Number of treated fingers		−1.73	−3.73 to 0.26	0.09
Needle fasciotomy vs surgery^a^		11.1	6.63 to 15.7	0.00
Needle fasciotomy vs collagenase		4.56	−0.05 to 9.17	0.06
Surgery vs collagenase^a^		−6.58	−11.1 to −2.90	0.00
**Retreatment**	**Intercept (Exponentiated**)	**OR**	**95% CI**	***P***
Age	0.75	0.97	0.92 to 1.02	0.23
Sex	0.09	1.35	0.48 to 3.81	0.57
Duration of contracture	0.12	0.97	0.91 to 1.04	0.43
Family history of Dupuytren contracture	0.10	0.97	0.41 to 2.30	0.95
Smoking	0.09	1.36	0.51 to 3.62	0.54
Baseline contracture	0.04	1.01	1.00 to 1.03	0.09
Little finger affected^a^	7.80	5.00	1.22 to 8.77	0.01
PIPJ dominantly affected^a^	0.00	2.79	1.20 to 6.48	0.02
Number of treated fingers	0.01	0.78	0.46 to 1.33	0.36
Diabetes	0.01	0.81	0.26 to 2.46	0.71
Alcohol consumption	0.00	1.04	0.99 to 1.09	0.11
Multivariable model	0.00			
Little finger affected^a^		4.23	1.40 to 12.78	0.01
PIPJ dominantly affected^a^		2.49	1.06 to 5.87	0.04
Needle fasciotomy vs surgery^a^		14.7	1.86 to 115	0.01
Needle fasciotomy vs collagenase		1.10	0.46 to 2.64	0.83
Surgery vs collagenase^a^		0.07	0.01 to 0.54	0.01

a Statistically significant.

The adjusted R^2^ value for the multivariable model for progression was 0.12. Treatment group contributed the largest proportion (47%) to the R^2^, followed by age (18%) and smoking (9%). Effect sizes were similar to those in the univariable models, but none of the variables—the little finger being treated, the number of treated fingers, or baseline contracture—remained statistically significant for progression once they were entered into the same model. These variables displayed multicollinearity (little finger involvement was associated with higher baseline contracture; *r* = 0.361), which inflated standard errors and widened the CIs, rendering the associations nonsignificant despite similar effect sizes to the univariable model (Table 3).

When fingers that underwent retreatment were included in the analyses, smoking was no longer associated with progression. Of the initially treated 24 fingers that received retreatment, 6 (25%) belonged to smokers, likely diluting the effect of smoking.

## DISCUSSION

This secondary finger-level analysis of DETECT found that surgery provides the most durable outcomes in terms of slower progression and lower need for retreatment in treated fingers compared with needle fasciotomy and collagenase. The benefit of surgery, however, was limited to treated fingers, and we observed no clinically relevant differences in progression in adjacent untreated or all initially untreated fingers. Younger age and smoking were associated with an increased risk of disease progression in treated fingers, and little fingers and fingers with the PIPJ being dominantly affected more often underwent retreatment compared with other fingers. Based on our model, a 50-year-old patient who smokes can expect 10 degrees greater disease progression in a treated finger over 2 years compared with a 60-year-old nonsmoker.

The between-group differences of 6 to 10 degrees in progression over 2 years are modest. Nevertheless, even small increments in angle may accumulate over time, and could accelerate reaching thresholds for recurrence, which is often defined as a 20-degree increase in contracture angle. We also examined the need for retreatment, which is an outcome with implications for both quality of life and health care costs. In our trial, fewer than 1% of treated fingers in the surgery group underwent retreatment within 2 years, compared with 8% to 9% in both percutaneous groups, highlighting a meaningful difference in durability.

Our findings align with earlier randomized controlled trials, which suggest that surgery results in lower recurrence rates compared with needle fasciotomy and collagenase.^21–28^ Whereas those studies measured recurrence as a dichotomous outcome using varying cutoffs of posttreatment contracture increase, we assessed progression as change in contracture angle or need for retreatment.

Consistent with our study, earlier studies have demonstrated that early onset age, typically defined as younger than 50 years, significantly increases the odds of recurrence.^29–31^ Moreover, involvement of the little finger, particularly the PIPJ, has been identified as a predictor of recurrence,^31–33^ and a weak association has been observed between higher baseline severity and increased recurrence risk.^32,33^ No association between smoking and disease progression was observed in earlier studies.

This study is limited by the relatively short 2-year follow-up, which may partially explain the low number of retreatments. However, ongoing follow-up up to 5 years will provide further insights into long-term outcomes. The low number of retreatments reduces statistical power to detect small effects in the retreatments, but analyses measuring progression in degrees yielded precise estimates excluding clinically relevant differences during the follow-up. Furthermore, surgical patients could only undergo surgery as a retreatment option, which may have reduced their likelihood of seeking retreatment in comparison with the other treatment groups. However, this approach reflects real-world clinical practice for these treatment strategies, although it may not apply in scenarios where different approaches are used. A further limitation is the exclusion of severely contracted fingers (>135 degrees), which may have led to an underestimation of recurrence and progression rates, as these fingers are likely at the highest risk for recurrence. However, this exclusion was necessary to ensure the safety and feasibility of all 3 treatment methods.

Fingers that underwent retreatment were excluded from the progression analysis to avoid underestimating progression due to the effect of retreatment. However, this exclusion may introduce survival bias, as fingers with faster progression were more likely to undergo retreatment and thus removed from the analysis. Because there were more retreatments in the percutaneous groups, this potential bias may disproportionately affect comparisons involving these groups. Nevertheless, sensitivity analyses that included fingers that underwent retreatment showed that the overall results were not substantially altered. The exclusion of fingers that underwent retreatment may also be one reason why involvement of the little finger was not associated with progression despite it being a predictor of retreatment.

The strengths of this study include the use of blinded contracture measurements and a pragmatic multicenter design, providing a representative sample. The finger-level analysis allowed for a detailed evaluation of disease progression not only in treated fingers but also in adjacent untreated and all untreated fingers, an aspect that has been largely unexplored in previous research and is uniquely addressed in this study.

## CONCLUSIONS

Surgery, needle fasciotomy, and collagenase injection provide comparable initial contracture release, but the effect of surgery is more durable in the treated fingers. Our data also indicate that disease progression in the adjacent untreated and all untreated fingers does not need to be considered when making treatment decisions for affected fingers. People with a higher risk for disease progression—young smokers with little fingers affected—may benefit the most from surgery in terms of slower progression and lower absolute risk of retreatment.

## DISCLOSURE

The authors have no financial relationships or conflicts of interest to declare. This study has been funded by the Research Council of Finland.

## Supplementary Material


