# Supplementary material for: Progression of Dupuytren Contracture: A Randomized Controlled Trial Comparing Surgery, Needle Fasciotomy, and Collagenase Injection
Source: Plast Reconstr Surg. 2025 Dec 4;158(1):101–12. doi: 10.1097/PRS.0000000000012666 (PMC13290052; doi:10.1097/PRS.0000000000012666)
Supplement: Supplementary file 1 [file prs-158-0101-s001.docx]

| **Residual contracture (°) in** | **3 months** | | | | | | **2 years** | | | | | | |
| --- | --- | --- | --- | --- | --- | --- | --- | --- | --- | --- | --- | --- | --- |
|  | **Mean (SD)** | | | **Mean difference (95% CI)** | | | **Mean (SD)** | | | **Mean difference (95% CI)** | | | |
|  | **Surgery** | **Needle fasciotomy** | **Collagenase** | **Needle fasciotomy vs. Surgery** | **Surgery vs. Collagenase** | **Needle fasciotomy vs. Collagenase** | **Surgery** | **Needle fasciotomy** | **Collagenase** | | **Needle fasciotomy vs. Surgery** | **Surgery vs. Collagenase** | **Needle fasciotomy vs. Collagenase** |
| **Treated**  **fingers** | 17 (19) | 14 (15) | 14 (18) | -0.8  (-6.8 to 5.1) | 0.5  (-5.4 to 6.3) | -0.4  (-6.4 to 5.7) | 16 (20) | 24 (25) | 20 (27) | | 9.7  (3.7 to 16) | -6.0  (-12 to -0.1) | 3.7  (-2.4 to 9.8) |
| **Adjacent fingers** | 2.3 (5.9) | 2.5 (5.6) | 5.2 (13) | 0.5  (-2.3 to 3.3) | -2.8  (-5.5 to -0.02) | -2.3  (-5.0 to 0.4) | 3.1 (9.6) | 2.1 (6.7) | 4.0 (10) | | -0.6  (-3.5 to 2.2) | -0.6  (-3.4 to 2.2) | -1.2  (-4.0 to 1.6) |
| **Untreated fingers** | 2.7 (11) | 1.9 (6.1) | 3.9 (11) | -0.1  (-1.9 to 1.7) | -1.1  (-2.9 to 0.7) | -1.2  (-3.0 to 0.6) | 4.0 (14) | 2.8 (9.6) | 4.9 (13) | | -0.5  (-2.3 to 1.2) | -0.9  (-2.7 to 0.9) | -1.5  (-3.3 to 0.3) |

**Table, Supplemental Digital Content 1.** Means and adjusted pairwise treatment effects for residual contractures at the three-month and two-year follow-up points.

SD = Standard deviation; CI = Confidence interval
